# High-stakes psychomotor ability assessment: a military selection case study of practice effects in airplane tracking tasks

**DOI:** 10.1186/s41235-025-00672-z

**Published:** 2025-12-03

**Authors:** Christopher Draheim, Ciara Sibley, Nathan Herdener, Aaron Cochrane, S. R. Melick, Kaylin Strong, Joseph T. Coyne

**Affiliations:** 1 Warfighter Applied Cognition and Technology Section, U. S. Naval Research Laboratory, 4555 Overlook, Avenue SW, Washington, D.C., 20375 USA; 2 Strategic Analysis Inc., Arlington, VA USA; 3 Brown University, Providence, RI USA; 4 Training Air Wing SIX, Pensacola, FL USA

**Keywords:** Psychomotor ability, Learning, Military selection, Aviation

## Abstract

Aviation selection tests are high-stakes assessments designed to identify candidates capable of succeeding in demanding flight environments. Most branches of the US military incorporate both content-based and process-based assessments to evaluate prior knowledge and reasoning ability, respectively. A challenge with high-stakes process tests is that their validity requires participant naivety, which is increasingly difficult to maintain in the modern internet era. As such, these high-stakes tests must be continuously evaluated to ensure the most valid, reliable, and cost-effective selection procedures are employed. To address this, we examined practice effects in the psychomotor airplane tracking tasks of the Navy’s Aviation Selection Test Battery (ASTB). We had 146 Naval Flight Students and 119 enlisted Sailors perform the ASTB’s psychomotor battery six times across two days. Results revealed large practice effects, shifting in rank ordering of individuals across attempts, and that psychomotor performance had not stabilized even by the sixth attempt. Prior action video gaming and flight simulator gaming experience correlated with psychomotor performance, with some evidence that improvements were related, albeit weakly, to either gaming experience or initial performance. Finally, correlations between psychomotor performance and eventual naval flight training scores were stable across the six attempts, but simulations indicated predictive validity can range widely if participants have differing levels of practice from one another. Overall, these findings indicate that the psychomotor component of the ASTB is a valuable inclusion to the Naval Flight School selection process but also could benefit from further refinement.

## Introduction

Individual differences in cognitive–perceptual abilities are of significant interest in the fields of education, industry, and military where ability tests are commonly used for personnel selection, placement, and advancement. The present study concerns individual differences in psychomotor ability (PMA) as it pertains to military aviation selection. Psychomotor testing has a long-standing tradition in the US military due to some of its unique characteristics and demonstrable predictive value. However, these unique characteristics also come with some challenges which our ongoing research aims to better understand and address.

## Fluid vs. crystallized abilities

An important consideration when using a cognitive test for personnel selection is how to balance the relative demands placed on crystallized knowledge vs. fluid reasoning, otherwise known as achievement vs. aptitude, respectively. Crystallized (achievement) tests capture the knowledge and skills the examinee has already learned, whereas fluid reasoning (aptitude) tests assess the examinee’s potential to learn to perform the desired role. In practice, tests will reflect both to some degree, but most lean more heavily into one or the other. On this front, many widely used selection test batteries have been criticized for overly emphasizing existing/acculturated knowledge (e.g., Burgoyne et al., 2021; Roberts et al., 2000). One reason for concern is that overreliance on existing knowledge results in an advantage for those who have higher education levels or otherwise more access to resources and opportunities to improve on the skill being assessed. Relatedly, individuals with more relevant background experience may perform better on the test independent of their eventual ability to perform the role being selected for. As such, some researchers argue that general selection tests should place more demands on fluid abilities to better capture an individual’s cognitive *potential* rather than the skills they have already acquired as a function of their access to education and other societal-related factors (e.g., Bosco et al., 2015; Burgoyne et al., 2021; Draheim et al., 2022).

Fluid-based selection tests are not without their own issues, however. In a review, Ackerman (2022) sought to assess whether “process” (fluid) or “content” (crystallized) intelligence tests were better predictors of academic success. He reported that his endeavor was more difficult than expected as he found himself in a proverbial house of mirrors with no definitive answer despite over a century of research to draw upon. He observed that two interrelated problems with fluid measures are their susceptibility to familiarity and learnability. Familiarity is a concern because fluid tests assume novel processing, i.e., that either the examinee is asked to perform something novel and/or that the stimuli are novel to them. To the extent that this is not the case, the examinee will likely perform above their true ability level. Learnability is the degree to which examinees have exposure to the test or specific items. Ackerman commented that learnability is an issue because examinees will improve their performance through practice, and even more so with practice involving instruction, coaching, and/or feedback. This can in turn reorder individual differences in test scores such that performance is not sufficiently reflective of the abilities and processes the tests were intended to measure, impacting both construct and criterion validity. Ackerman also observed that between-subject performance variance typically decreases with practice and learning, resulting in smaller correlations with post-practice performance on fluid tests to other variables of interest, such as general intelligence. This reduction of variance poses a potential issue for using tests for selection if examinees are well practiced on them.

Familiarity and learnability pose significant challenges to modern *high-stakes testing* in which performance has real-world implications for the examinee. Most high-stakes ability tests assume respondents are reasonably naive to test content, demands, and strategies required for success. This assumption is critical for maintaining construct and criterion validity because when familiarity with the test increases for a subset of test takers (whether through repeated access to the actual test or test materials) the test may capture different constructs for different individuals. Thus, the main advantage of fluid tests over crystallized ones, that they are a better indicator of capacity to learn, is lost if the test is not novel to some respondents due to previous examinees sharing test content, strategies, and even unofficial practice emulators with motivated prospective examinees. To combat this issue, most modern cognitive ability tests are proprietary with closely guarded items. Additionally, test forms are subjected to continuous large-scale data collection, norming, and scaling efforts on thousands of items which enables alternative forms and adaptive testing. This means that examinees receive different items and, importantly, ones they are very unlikely to have encountered before in their test preparation or even previous administrations of the test. However, this is not true of all selection test batteries which may consist of non-adaptive subtests with a small pool of items or trial-based tasks in which examinees perform a handful of similar trials. For these tests, it is critical to understand how potential familiarity and learnability may impact examinee performance and test validity.

The present research concerns individual differences of psychomotor abilities specifically within the context of US military aviator selection. The following sections will provide a historical overview of PMA and its use in military selection and then return to some of the aforementioned issues as they pertain to the test battery of the present interest.

## Overview of psychomotor ability

### Psychomotor vs. cognitive ability

As with many psychological constructs, a precise definition of PMA is difficult to offer due to a lack of consensus among researchers (e.g., Chaiken et al., 2000), but a hallmark feature is that psychomotor tests place demands on both perceptual processing *and* the motor response, whereas tests of more traditional cognitive abilities (e.g., intelligence; memory; processing speed) primarily tax perceptual–cognitive processing with minimal response demands. In other words, psychomotor tests have a more involved and complex muscular–motor response than the standard cognitive test, and typically with smaller demands on higher-order cognition (c.f., Ackerman, 1988). Another feature is that psychomotor tests are generally quite amenable to practice and training as one progresses through skill acquisition phases. For modern real-world examples, consider how much emphasis is given to deliberate and extensive practice for performing at a high level in musical and athletic endeavors (which involve a large degree of psychomotor skills), or that most young adults become proficient automobile drivers within their first couple years of receiving their license after hundreds of hours of practice.

Whether PMA is a cognitive ability has not always been clear. Many lines of research suggest that psychomotor skills are largely independent of cognitive ones. For example, PMA is sometimes considered synonymous with simple motor ability and hand–eye coordination (e.g., Ostoin, 2007), and PMA has not traditionally been an included component in models of human cognition and intelligence (e.g., Carroll, 1993). But, as Fitts (1954) intuited, studying the human motor system isolated from sensory mechanisms is not possible and so psychology of the motor system is necessarily in the realm of perceptual–motor and thus cognitive to some degree. Further, the seminal Cattell–Horn–Carroll three-stratum hierarchical model of human abilities/intelligence (Carroll, 1993) has been revised and updated to now include psychomotor-based abilities (see Flanagan & Dixon, 2013; Flanagan & McGrew, 1997; McGrew, 2005, 2009; and Schneider & McGrew, 2012). Specifically, early revisions to the model included PMA as a second-order factor (Gps) interpreted as “broad psychomotor speed” which concerned the speed of digit and limb movements independent of processing and perceptual speed abilities. In subsequent revisions, a separate PMA factor (Gp) was added to the model which included a number of traditional (and not necessarily speeded) psychomotor subfactors previously outlined by Fleishman (1954, 1964) such as finger dexterity, arm–hand steadiness, and multilimb coordination. Although PMA was not typically assessed on cognitive tests and is often considered separate from intelligence, Flanagan and McGrew argued that the recognition of broader psychomotor abilities within the Cattell–Horn–Carroll model was warranted because individual differences research revealed a relationship between PMA and general intelligence, the influence of PMA in modern intelligence batteries, and the importance of perceptual–motor functions in neurological assessments.

Regardless of where PMA is placed in models of cognitive ability, some psychomotor tasks do clearly involve cognitive processing. For example, they may have a continuous nature with a multitasking or task switching component, and some also often require the respondent to estimate time either throughout the task or specifically regarding timing a response at a critical point in the task (Chaiken et al., 2000). As such, another way to conceptualize psychomotor tasks is thus that they vary along two dimensions: complexity of stimulus (i.e., degree of perceptual–cognitive processing required) and complexity of motor activity required for responding (Chaiken et al., 2000; Fleishman, 1953).

### Psychomotor testing for personnel selection

Psychomotor abilities have been of interest to employers and militaries dating back at least around a century. This is perhaps because psychomotor measures not only assess some degree of perceptual–cognitive ability, but they also assess motor coordination and the ability to integrate environmental input, cognitive processing, and motor outputs. This complex set of skills is required for many important jobs and roles in modern society, from more routinized ones such as mechanical repair and ordinance work, to ones involving making motor outputs while also processing and integrating complex sensory information such as driving, piloting an aircraft, and operating complex machinery. Broadly speaking, PMA is also needed for body and motor control to assist with the movement, coordination, dexterity, flexibility, and speed of physical actions and decisions. To this end, Paśko et al. (2022) stated that PMA is, “…crucial for the soldiers to be able to quickly and efficiently make effective decisions under stressful situations” (p. 1); Prokopczyk and Wochyński (2022) argued that specialized PMA training should be given to pilots as part of their skill preparation because of the importance of habituation and automatization of psychomotor skills in flying an advanced aircraft; and Chaiken et al. (2000) stated that PMA underlies a diverse set of activities ranging from driving, playing video games, surgery, electrical repair, operating a computer, and all types of athletic performance and physical endeavors. It has also been argued that psychomotor skills are a main learning outcome in essentially all procedure-oriented professions (Changiz et al., 2021; Thoirs & Coffee, 2012).

Regarding real-world applications, PMA has been shown to be predictive of criterion performance in a variety of fields and occupations ranging from simple to complex, including manual jobs (see Hunter, 1980 for a large-scale review), soldier marksmanship (Anglin et al., 2017), air traffic controllers (Ackerman, 1988), surgeons (Hofstad et al., 2012), and military pilots (Carretta & Ree, 1994). Importantly, psychomotor tests have been shown to predict individual differences in both skill acquisition and criterion performance even after extensive practice (Ackerman & Cianciolo, 2000; Fleishman, 1956). Psychomotor tasks have therefore continued to have a role in US military selection because of this predictive validity and also because they tap into motor-related unique performance variance that is difficult or otherwise impossible to capture with more traditional perceptual–cognitive tests. The present research more narrowly focuses on PMA as it relates to selecting US Naval Flight Students, although the results should be more broadly relevant to using PMA for selection purposes in military and perhaps other settings.

## Psychomotor ability and US military aviation: a brief review

The US military began employing aviators at large scale at the start of World War I when droves of recruits volunteered to take to the skies, which necessitated the establishment of flight training centers (Griffin & Koonce, 1996). It soon became clear that pilot casualties during the war were not just the result of combat and equipment failures, but were also due to human error with alarming regularity, something that remains true in modern military and commercial aviation. This prompted widespread efforts to develop and employ prediction tools to identify the individuals who were most likely to succeed as aviators. In addition to physiological, emotional, and perceptual–cognitive tests, the US military took an interest in apparatus-based psychomotor tests for this purpose. A unique challenge that subsequently arose in World War II was the urgent need for the USA to train large numbers of pilots in a short period of time, thus significantly raising training costs and placing more importance on accurate selection (Griffin & Koonce, 1996). The Department of Defense was therefore more willing to use expensive and cumbersome apparatus-based tests if they might improve the selection process. For example, one of the most popular psychomotor tests of this era was the Mashburn “complex coordination” task requiring multilimb coordination ability closely simulating the stick and rudder movements of an airplane. Contrasted with traditional pencil-and-paper ability tests, this test, like many apparatus-based tests, was expensive to build and maintain, required constant and careful calibration, required trained examinees, and did not afford simultaneous group or large-scale testing of examinees (e.g., Ackerman, 1997; Fleishman, 1953; Melton, 1947). Even so, the US Air Force (née Army Air Corps) used it and roughly a dozen other apparatus-based psychomotor tests during the war as part of their Aircrew Classification Test Battery which determined whether an applicant was most suited to the role of pilot, navigator, bombardier, or gunner (Melton, 1947).

Proper criterion validity of pilot selection tools was difficult before and during the initial stages of World War II for a variety of reasons (see Griffin & Koonce, 1996), but this began to improve during the war along with postwar analysis and insights. Some of these analyses revealed that physiological measures had virtually no validity for pilot selection whereas apparatus-based psychomotor tests provided decent incremental validity to standard paper-and-pencil perceptual–cognitive tests. Another insight was that both cognitive and psychomotor tests were strong predictors of initial training success but were relatively poor at predicting success in advanced operational training or eventual combat proficiency (McFarland, 1953).

In the early 1970s, automated replicas of limb coordination psychomotor tests were developed, including the Mashburn complex coordination task. Follow-up analyses demonstrated they were significant predictors of Air Force training success (Griffin & Koonce, 1996; Sanders et al., 1971). By 1988 these tests had been programmed for the personal computer and Kantor and Carretta (1988) showed that they significantly predicted pass–fail flight training rates in the US Air Force Flight Training program, predicting roughly 20% of the variance when combined with perceptual–cognitive tests. Other researchers also found that psychomotor tests provided around 15% incremental validity in predicting flight school performance above and beyond other cognitive tests (e.g., Delaney, 1992; Griffin & Koonce, 1996). The automation of psychomotor tests provided the opportunity to include them in the newly automated Aviation Selection Test Battery (ASTB), which was a revision and relabeling of a test battery used since World War II to predict initial performance in ground school and primary flight training in the US Navy.

At present, the ASTB is used by the US Navy, Marine Corps, and Coast Guard to screen and select aviation officer candidates. The US Air Force also uses the Pilot Candidate Selection Method score as part of their selection process and two of the three components to this score, the Air Force Officer Qualifying Test and the Test of Basic Aviation Skills, are from test batteries which, in aggregate, are very similar to the ASTB. The ASTB has been shown to predict relevant flight training outcomes such as academic and flight training scores, academic setbacks, and attrition (Navy Aerospace Medical Institute, 2024; also see Sibley, 2024). One internal analysis estimated that it saved just the US Navy over 42 million USD in attrition cost avoidance in 2013 alone, which was a reduction of roughly 40% relative to not using it (Moclaire et al., 2014). This number has since been updated to $52 million on the official ASTB frequently asked questions page (Navy Aerospace Medical Institute, 2024) and is likely higher than that today.

## The performance-based measurement battery (PBM)

Today, psychomotor testing for US military aviators is implemented through the Performance-Based Measurement Battery (PBM), which has been a component of the ASTB^1^ since 2013. In civilian participants, a recent study by Mashburn et al. (2024) demonstrated that PBM performance correlated strongly with simulated work performance.^2^ In operational settings, the PBM has been shown to provide meaningful incremental prediction for Naval Flight School performance above and beyond the math skills, reading comprehension, and mechanical/aviation/nautical comprehension tests that are assessed in other ASTB subtasks (e.g., Nye et al., 2020; Phillips et al., 2011).

The PBM is a group of timed tests assessing abilities such as hand–eye coordinated tracking, attention, task switching, decision making, and spatial ability. It is performed with a hands-on throttle-and-stick (HOTAS) joystick-like attachment that simulates aircraft controls, with examinees using their left hand for the throttle and their right hand for the stick. The first four subtests are (in order) direction orientation, dichotic listening, vertical airplane tracking (VTT; performed with the left-hand throttle), and two-dimensional airplane tracking (ATT; performed with the right-hand stick), all performed in isolation. Examinees then perform the two tracking tasks simultaneously (AVTT), then they perform the AVTT along with a dichotic listening test (multitracking task), and finally they perform the AVTT with emergency scenario prompts. The tracking demands of the psychomotor tests in the PBM correspond to the rate control subfacet of PMA, which was defined by Chaiken et al., (2000; also see Fleishman, 1954) as the ability to perform sustained attention to continually correct motor responses to either keep synchrony with a variably speeded object or to avoid collisions with an object.

Of note is that while the PBM’s HOTAS attachment increases the cost and upkeep demands of administering it relative to a more standard computerized task only requiring a mouse and a keyboard, it is still a relatively affordable option that avoids some of the historical challenges with apparatus-based tests such as requiring trained experimenters, requiring equipment requiring frequent calibration, preventing group testing, and so on.

### Selection challenges with the PBM

Although the PBM has been an important component of the ASTB since its implementation, there are still a number of questions with the battery as well as potential areas for improvement that warrant study. Some of these include challenges pertain to psychomotor tests more generally and are compounded with the PBM because it is part of a high-stakes test battery used to select and place individuals into highly desirable roles within the US military (i.e., aviation officers). We will describe these issues in some detail before turning to the primary motivation and research questions for the present study.

The ASTB, including the PBM, consists of the same subtests that are only occasionally re-normed and updated, and with high-stakes testing in the modern internet era the assumptions about examinee naivety are increasingly tenuous. Unofficial preparation advice, including ASTB test items, strategy suggestions, and even high-quality PBM replicas are available in online military pilot communities, resulting in some portion of the prospective candidate pool being aware of the PBM’s test content. And because the ASTB is used as a selection test for highly desirable and competitive roles within the US military, applicants are especially motivated to seek out advantages on the test, and thus, many have extensive practice on the PBM prior to officially taking it.

As discussed at the onset of this paper, it can be problematic in selection testing if participants take them with differing levels of experience and familiarity with the test, as this can negatively impact its psychometric properties: namely reliability, construct validity, and criterion validity. As a case example of this, consider the current PBM spatial subtest called the direction orientation task. During the PBM’s development and validation prior to it being added to the ASTB in 2013, in-lab validation studies showed the direction orientation task significantly predicted flight training performance. However, previous examinees had shared so much information online with prospective applicants that within just three years the test no longer correlated with training performance and its validity as a measure of spatial ability has also called into question (see Coyne et al., 2022). This seems to be because practice effects on the test are particularly strong and motivated individuals can find information online that includes simple mathematical and other non-spatial strategies to perform the test. Consequently, there have been large annual increases in scores and the majority of candidates get at most one question wrong, impacting predictive validity. One specific concern with the PBM is that because there are no official practice opportunities for its subtasks, prospective applicants must seek out unofficial test information, content, and practice on their own. As such, some applicants will be simply unaware that unofficial test preparation guides and task simulators are available, and that others who are part of the aviator in-group will likely be more aware of these opportunities. Further, individuals with less access to resources and free time will not only be less aware of potential test preparation options but also may not have the ability to devote as much time to practicing as others.

Importantly, one subtest losing validity over time does not invalidate the entire test battery as selection decisions are based on weightings of all the subtests which can be adjusted accordingly, but nevertheless underperforming subtests in a selection battery are a potential waste of resources and also provide opportunities to improve the selection process if replaced with stronger tests. An important element of personnel selection testing is therefore to continue to evaluate and monitor operational tests, identify potential shortcomings, and address them with modifications or replacement as necessary.

For the PBM specifically, subjective reports from our unpublished data indicate that applicants believe a major aspect of improving on the task is becoming comfortable with the HOTAS device used to perform it, especially as the y-axis for the two-dimensional tracking task (ATT) is inverted such that moving the stick forward moves the cursor down and pulling the stick back moves the cursor up (also see Draheim et al., 2024). This is an additional barrier that could contribute to performance differences if prospective applicants do not have access to or otherwise cannot afford purchasing a HOTAS (as of July 2024 the HOTAS used to perform the PBM is listed at 249.99 USD on Logitech’s website and 228.88 USD on Amazon.com). It may also create an advantage for people who have more experience with similar controllers from video games, especially flight simulators or action games that often implement inverted axis controls. It is therefore not surprising that both HOTAS experience and video gaming experience positively correlates with PBM performance (Drollinger et al., 2015).

To summarize the present concerns with the PBM: (1) psychomotor tasks are highly trainable and show large practice effects, which can be a challenge to selection if examinees come in with differing levels of test experience and familiarity, (2) background variables which presumably are irrelevant to whether one is capable of becoming a good pilot (i.e., construct-irrelevant variance), including video gaming experience and HOTAS experience have been shown to correlate with PBM performance; and (3) both of these concerns are potentially magnified with the PBM because it is a high-stakes test and no official details test information or practice opportunities are provided for it. As such, some prospective applicants are able to find test information, strategy advice, and even high-fidelity simulators to practice through online communities and/or their social connections.

## The present study

Motivated by the concerns outlined above, we sought to better understand the nature of practice effects in the PBM. We were especially interested in the magnitude of practice effects in both an aviation sample and a sample of enlisted individuals who were presumably naive to the ASTB, and the extent to which practice among aviators affected the predictive validity of eventual flight school performance. Additionally, we planned to assess practice effects and predictive validity when considering relevant action video gaming and flight simulator experience. Our goal was to use the insights gained from this study to provide a more comprehensive evaluation for ASTB policy makers and potentially offer guidance for future revisions of the PBM to improve its predictive validity.

We had both enlisted and officer Navy personnel perform an in-house recreation of the PBM six times over the course of two days (i.e., three attempts on each of two days). Participants also completed questionnaires regarding various background variables, including relevant gaming experience. Our design was set up to address three sets of interrelated research questions:

1. To what extent does performance improve over time with practice on psychomotor tracking tasks? How stable is rank ordering of individuals during practice?

### H1a:

 Participants will improve substantially on the two tracking tasks. Further, enlisted Sailors, who are naive to the PBM, will exhibit larger overall performance gains than Naval Flight Students (NFS), who have previously taken the PBM and were selected partly based upon their passing scores.

### H1b:

 Independent of group membership, lower initial performers will improve more across PBM attempts relative to higher initial performers. H1c: Test–retest reliability will decrease across PBM attempts for both tracking tasks, as will the correlation of in-laboratory PBM performance with participants’ scores on the official PBM, indicating meaningful shuffling in the rank ordering of individuals.

### H1c:

 Participants will have reached asymptotic or near-asymptotic levels of performance by the sixth attempt of each tracking task.

2) Do individuals differentially benefit from PBM practice based on relevant experience with video games, including flight simulator games?

### H2:

Participants lacking relevant gaming experience will improve more quickly relative to those with more experience.

3) To what extent is the tracking tasks’ prediction of flight school performance impacted by practice?

### H3:

 Because prior experience should be unrelated to whether someone can eventually succeed in flight training, we hypothesize that providing practice opportunities should help level the playing field and thus improve the PBM’s predictive validity.

To summarize the research questions into one overarching aim of the study, we endeavored to provide sufficient practice for participants to approach their individual performance ceiling and then assess whether PBM scores would remain predictive of eventual flight school success and potentially even show improved predictive validity due to practiced scores being a better representation of a person’s true ability level.

## Method

### Participants

A total of 461 individuals participated in the first session. However, 183 did not return for the second session a week later due to scheduling challenges inherent to working with active duty populations and an additional 13 participants were removed for reasons explained in the Data Processing and PBM Scoring subsection below, resulting in a final *N* = 265 (25 women). Failure to return was primarily due to administrative factors outside participant or experimenter control and checks of the data confirmed it was not reflective of meaningful group differences between returning and non-returning participants.

In the final sample, 146 were US NFS, meaning they were Naval officers selected to become either a pilot or flight officer, who had not yet begun their military flight training. The remaining 119 were enlisted US Navy personnel who were enrolled in a variety of aviation support training programs such as Aviation Mechanic, Aviation Ordnance, and Aircrew. The mean age was 23.7 years (*SD* = 3.4) with 76% reporting some college experience. All participants provided informed consent prior to beginning the experiment, and the study’s protocol was approved by Naval Research Laboratory’s Institutional Review Board (protocol NRL.2021.0003). Participants were volunteers who were not directly compensated but were informed that their participation may help improve personnel selection within the US military.

### Procedure

Data collection took place at US Naval Air Station in Pensacola, FL, over two days which occurred one week apart at the same time of day. Each session involved three attempts of the psychomotor portions of the PBM as described in the following section, for a total of six attempts. Data were collected in a room equipped with 20 computer stations separated by dividers to obscure the view of other participants and their screens. Each station was equipped with a computer with a Windows operating system, 24-inch monitor set to 2560 × 1440 resolution at 60 Hz, a Logitech X52 HOTAS, and headphones. Participants arrived in groups of up to 20 individuals and were given detailed verbal instructions about the series of tasks that they would be completing. Both sessions took approximately three hours each, for a total participation time of roughly six hours per participant.

In both sessions, participants received instructions for calibrating an eye tracker and then performed two simple eye tracking tasks that are not discussed here. Next, participants were provided instructions for logging onto the official Navy test site and accessing a practice version of the PBM. Participants were informed that they would be completing this test three times consecutively and would receive their score after each round. Participants were instructed to write their score on a piece of paper after each PBM attempt and wait until everyone else had finished. To enhance motivation and engagement, after the group had finished each attempt the experimenter would discuss current high scores and determine whether anyone had beat the current high score for their specific military rating (e.g., Flight Student, Aircrew). After the third PBM round of the day, participants were asked to complete demographic and gaming experience surveys (day 1) or other questionnaires not discussed here (day 2), followed by a series of cognitive tests not relevant to the present study.

#### Iterative PBM

**Fig. 1:**
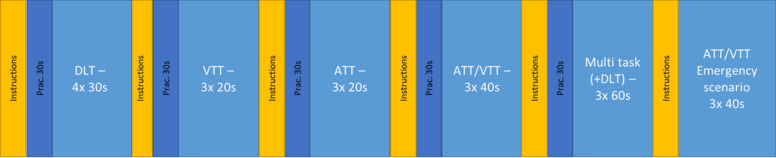
Timeline of One Attempt on the Iterative PBM. *Note*. One complete round through our in-laboratory iterative PBM, including self-paced instructions and 30 s of unscored practice preceding each subtask (except for the emergency scenarios test). All unscored practice was removed and the instructions were condensed for the 2nd and 3rd attempts in each session (i.e., attempts 2, 3, 5, and 6)

In collaboration with the ASTB software developers and with approval from the Naval Aerospace Medical Institute in Pensacola, FL who control the ASTB/PBM, our team at the US Naval Research Laboratory created a version of the PBM that included three rounds of all its subtests except for the direction orientation test (which was excluded from the present study as it is not relevant for psychomotor investigations). This iterative PBM was administered as a full-screen application via a secure web-based software platform using the Mozilla Firefox browser. It had modified instructions for improved clarity, and delivered condensed summaries of instructions for the second and third attempt of both days. Following the design of the official PBM, instructions were self-paced and the first attempt of each day (i.e., attempts one and four) included 30 s of unscored practice for each subtest except for the emergency scenarios test. Further, the airplane target would redirect with randomized movements when successfully tracked for a period of time. Figure 1 shows the progression of the first round and their durations, and the following subsection describes these tasks in more detail. Also see Phillips et al. (2011) and Walker et al. (2007) for more details on the PBM and its subtasks.

### Iterative PBM subtasks

#### Dichotic listening test

Performance on this test was not of interest for the present study and performance data on it were not analyzed, but it was administered because it is a component of the multitracking task. This task requires that respondents wear stereo headphones and listen to numbers and letters simultaneously presented in both ears while their left hand is placed on the throttle and right hand is on the stick. Before a set of trials they were told which ear to attend to and instructed to respond by depressing the stick button when an even number is presented in the target ear, and to depress the throttle button for an odd number. The task lasted for 120 s.

#### Vertical tracking task (VTT)

This task was performed with the throttle in the respondent’s left hand and required that they track, with a red crosshair, a yellow aircraft as it moved unpredictably up and down the left side of the screen. Respondents performed the task for 60 s. The target increased in speed every 20 s and also adaptively redirected with random movements if the respondent was successfully tracking it for a period of time.

#### Two-dimensional airplane tracking task (ATT)

This task was performed with the stick in the respondent’s right hand and required that they track, with a red crosshair, a yellow aircraft as it moved unpredictably across the screen in horizontal, vertical, or diagonal lines. The stick’s y-axis controls were inverted such that moving it forward moved the cursor down on the screen and vice versa. As with VTT, respondents performed the task for 60 s and the target increased in speed every 20 s and also adaptively redirected with random movements if the respondent was successfully tracking it for a period of time.

#### Concurrent airplane and vertical tracking task (AVTT)

**Fig. 2:**
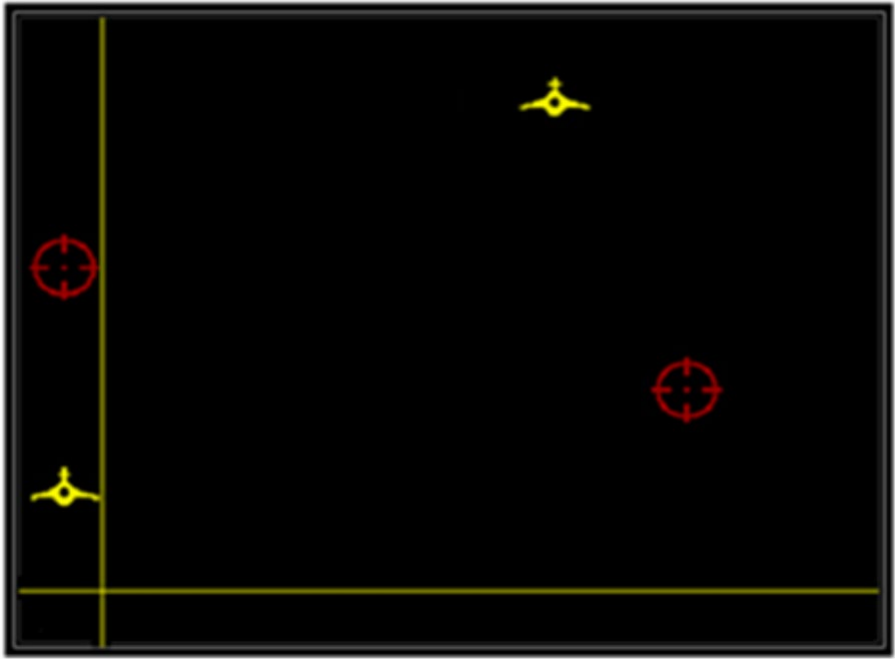
Image of the AVTT

Respondents performed the ATT and VTT simultaneously using the throttle in their left hand and the stick in their right hand. Respondents performed the test for 120 s with the speed of both targets increasing every 40 s. Figure 2 shows a screenshot of this task with the VTT on the left part of the screen and ATT filling the rest of the screen.

#### Multitracking task

Respondents performed the AVTT and dichotic listening test simultaneously for 180 s with the speed of both targets increasing every 60 s.

#### Emergency scenario test

Respondents performed the AVTT for 120 s while also periodically receiving emergency scenario prompts that they were informed about during the instructional phase and given a specific series of button and knob responses to memorize and execute as quickly as possible when prompted. A total of three prompts occurred throughout the test.

#### Demographic and gaming survey

After finishing the three rounds of the iterative PBM in the first session, participants completed a computer-based survey regarding demographic information and potentially relevant experience with video games, aviation, flight simulators, and flight equipment. For the present purposes, we were most interested in action video gaming experience and experience with flight simulator games. For action gaming and flight simulator experience, the survey asked respondents to report both their childhood (aged 3–13 years) and current (within the last 12 months) levels of engagement. We analyzed data from both childhood and adult (i.e., current) separately, as previous studies have found differential relationships with childhood vs. current gaming and PBM performance (e.g., Drollinger et al., 2017).

For video game experience, the survey contained a modified version of the Bavelier Lab Video Game Questionnaire (e.g., Brain & Learning Lab, 2024; Green et al., 2017) which asked respondents to report “…the number of hours played per week when you played the most” across nine different gaming genres: “First/Third-Person Shooters,” “Action-RPG/Adventure,” “Sports/Driving,” “Real-Time Strategy/MOBA,” “Flight Simulation Games,” “Turn-Based/Non-Action Role-Playing/Fantasy,” “Turn-Based Strategy/Life Simulation Puzzle,” “Music Games,” and “Other.” Popular games were provided as examples within each category. There were six response options for each question: “Never,” “Less than one hour,” “Between 1 and 3 h,” “Between 3 and 5 h,” “Between 5 and 10 h,” and “More than 10 h,” which we coded as 0–5, respectively, for data analysis.

#### Action video gaming

We summed the scores from the first four video gaming categories (First/Third-Person Shooters, Action-RPG/Adventure, Sports/Driving, and Real-Time Strategy/MOBA) to create a variable representing action video gaming experience, again with separate scores for childhood vs. current gaming frequency. Given responses from each individual question were coded as 0–5, the aggregated action gaming engagement score could range from 0 to 20.

#### Flight simulator experience

Flight simulator experience was taken from the video gaming survey and ranged from 0 to 5, with separate scores for childhood vs. current (i.e., adult) engagement.

## Results

### Data processing and PBM scoring

Data analyses were conducted with a combination of R Statistical Software (R Core Team, 2024), JASP (JASP Team, 2024), and SPSS.

We scored PBM performance according to the scoring method used for the official version of the test, which is done with task-specific composite scores that weigh behavioral performance for the VTT, ATT, dichotic listening test, and emergency scenario test across the various subtasks. For example, the ATT composite score is a weighted aggregate of performance on the ATT when performed by itself, the ATT component of the AVTT, the ATT component of the multitracking task, and the ATT component of the emergency scenario test. Calculation and weighting of the composite scores are proprietary, but for VTT and ATT they correlate strongly with the amount of time the cursor is over the target. Henceforth, all mention of VTT and ATT performance refer to their respective composite scores unless noted otherwise. See Phillips et al. (2011) for more details on scoring methodology.

We removed 13 individuals from all subsequent analyses because their VTT or ATT performance was more than three standard deviations below the mean on at least half of the attempts, suggesting lack of engagement with the task. This resulted in a final dataset with *N* = 265 participants (119 enlisted; 146 NFS).

### Research question 1: effect of repeated PBM attempts

#### Rate of improvement

Descriptive statistics for aggregate data are provided in Table 1 which shows performance and gains on the VTT and ATT across attempts. As a check of the data, we compared mean scores and SDs from the 44,000 individuals for whom we had official PBM data (VTT *M* = 46.69, *SD* = 15.70; ATT *M* = 32.75, *SD* = 18.77; see Table 5 of Sibley, 2024) and found that scores were comparable to scores for participants in the present study. This provided evidence that our participants were motivated and of roughly equivalent ability to prospective candidates taking the ASTB.

**Table 1:** Descriptive statistics and cumulative improvements for VTT and ATT

Task	Attempt	*M*	*SD*	Skew	Kurtosis	Cumulative Gains (*d*)
VTT	1	35.90	10.49	0.80	1.49	–
	2	41.30	11.46	0.48	0.65	0.51
	3	44.23	12.42	0.58	0.46	0.79
	4	46.04	13.12	0.31	0.51	0.97
	5	49.12	12.83	0.59	0.71	1.26
	6	50.22	13.08	0.32	0.53	1.37
ATT	1	26.07	15.21	0.19	−0.87	–
	2	33.15	16.70	0.09	−0.60	0.47
	3	36.62	16.71	0.12	−0.53	0.69
	4	38.08	16.86	−0.07	−0.61	0.79
	5	42.70	17.69	−0.21	−0.47	1.09
	6	44.16	18.12	−0.10	−0.45	1.19

*N* = 265. The dependent variable for VTT and ATT are their proprietary composite scores which weigh behavioral performance across each instance of them throughout the PBM. Cohen’s *d* gains are cumulative (i.e., compared to the first attempt) and were calculated using the first attempt’s standard deviation as the denominator for standardization

Except for 1.49 kurtosis for VTT attempt one (i.e., the first task participants encountered in this study), all skew and kurtosis values were within ± 1, indicating an acceptably normal distribution of scores. Overall improvements from attempts one to six were statistically significant for both tasks. In terms of Cohen’s *d* gains (SD standardized from the first attempt), improvements were around *d* = 0.50 on the 2nd attempt for both tasks, and for attempt six were cumulatively *d* = 1.37 for VTT and *d* = 1.19 for ATT.

**Fig. 3:**
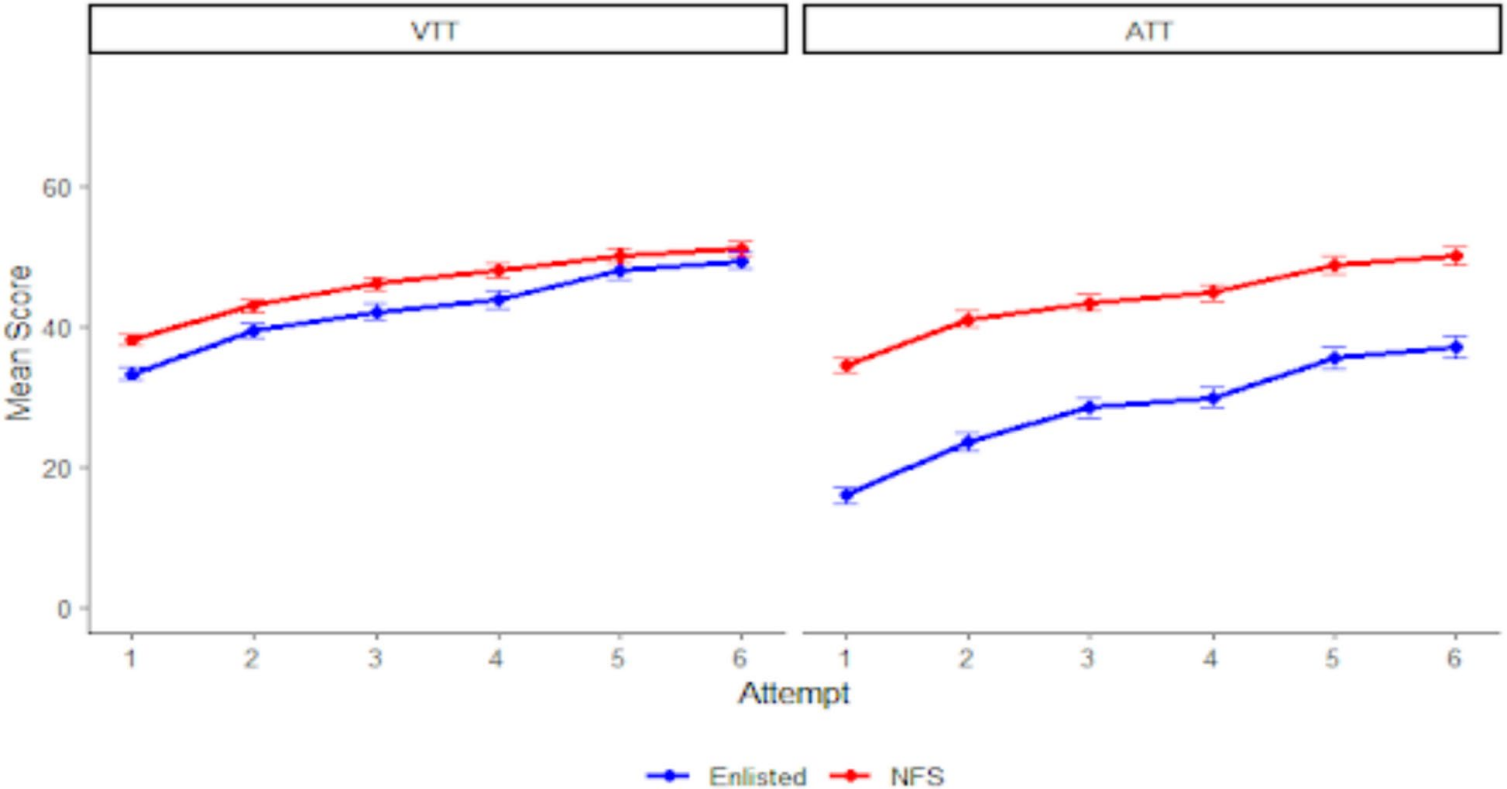
VTT and ATT Performance by Group Membership. *Note*. *n* = 146 for NFS, *n* = 119 for enlisted. Error bars represent standard error of the mean

Table 2 shows descriptive statistics and improvements broken down by group membership (enlisted personnel vs. NFS). For VTT, enlisted personnel improved a total of *d* = 1.56 and NFS improved *d* = 1.27 from attempts one to six. For VTT, cumulative improvements were *d* = 1.80 for enlisted and *d* = 1.25 for NFS. Both groups also experienced the largest gains between the first and second attempt (see Fig. 3); *d* = 0.66 for enlisted and *d* = 0.53 for NFS. Of note is the much larger score gap between enlisted and NFS on the ATT relative to the VTT.

**Table 2:** Gains by group membership

Task	Attempt	Enlisted					NFS				
		*M*	*SD*	Skew	Kurtosis	Cumulative Gains (*d*)	*M*	*SD*	Skew	Kurtosis	Cumulative Gains (*d*)
VTT	1	33.23	10.28	0.76	1.24	–	38.08	10.17	0.96	1.82	–
	2	39.28	11.69	0.40	0.16	0.59	42.95	11.04	0.63	1.05	0.48
	3	42.01	12.67	0.69	0.64	0.85	46.04	11.96	0.57	0.41	0.78
	4	43.79	13.58	0.38	0.41	1.03	47.87	12.47	0.35	0.64	0.96
	5	47.88	13.10	0.73	0.74	1.43	50.13	12.56	0.48	0.73	1.18
	6	49.31	13.32	0.59	0.57	1.56	50.97	12.88	0.09	0.58	1.27
ATT	1	15.91	11.70	1.29	2.62	–	34.35	12.49	−0.35	−0.25	–
	2	23.59	14.67	0.85	1.25	0.66	40.94	14.02	−0.29	−0.16	0.53
	3	28.36	15.27	0.70	0.62	1.06	43.34	14.73	−0.18	−0.24	0.72
	4	29.84	16.05	0.51	0.17	1.19	44.80	14.38	−0.37	−0.22	0.84
	5	35.43	17.58	0.27	−0.15	1.67	48.62	15.48	−0.54	0.09	1.14
	6	37.02	18.15	0.28	−0.08	1.80	49.97	15.93	−0.27	−0.35	1.25

*n* = 146 for enlisted; *n* = 119 for NFS. Cohen’s *d* gains are cumulative (i.e., compared to the first attempt) and were calculated using the first attempt’s standard deviation as the denominator for standardization

Separately for both VTT and ATT performance, we conducted a linear mixed-effects model to examine the effects of repeated practice (PBM attempt number), group membership (enlisted or NFS), and their interaction. Participant was included as a random effect to account for repeated measures.

**VTT.** The VTT model revealed significant main effects for attempt, *F*(5, 1315) = 235.36, *p* < 0.001, η_p_^2^ = 0.47, and group membership, *F*(1, 263) = 6.33, *p* = 0.012, η_p_^2^ = 0.02, indicating composite scores varied significantly across attempts and by group. The interaction between attempt and group membership was also significant, *F*(5, 1315) = 3.03, *p* = 0.010, η_p_^2^ = 0.01, indicating that the effect of practice depended on group membership.

Post hoc pairwise comparisons using Tukey-adjusted contrasts were performed to compare performance between attempts, within each group. Estimated marginal means significantly increased across each consecutive attempts for both groups, except for attempts three to four, *t*(1315) = 2.43, *p* = 0.146 for enlisted; *t*(1315) = 2.77, *p* = 0.064 for NFS, and attempts five to six, *t*(1315) = 1.94, *p* = 0.376 for enlisted; *t*(1315) = 1.26, *p* = 0.806 for NFS. Interestingly, NFS performed significantly better than enlisted on the first four attempts, but not attempts five, *t*(388) = 1.50, *p* = 0.136, or six, *t*(388) = 1.10, *p* = 0.271.

Overall VTT improvements across the six attempts were large for both groups (see Table 2). In terms of Cohen’s *d* improvements, enlisted participants improved a total of *d* = 1.56 and NFS participants improved *d* = 1.27. Both groups also expectedly experienced the largest gains between the first and second attempt, *d* = 0.59 for enlisted and 0.48 for NFS.

**ATT**. The model for ATT performance also showed significant main effects for attempt, *F*(5,1315) = 378.61, *p* < 0.001, η_p_^2^ = 0.59, and group membership, *F*(1, 263) = 76.34, *p* < 0.001, η_p_^2^ = 0.22, as well as a significant interaction effect, *F*(5, 1315) = 10.10, *p* < 0.001, η_p_^2^ = 0.04, indicating that gains depended on group membership.

We conducted post hoc pairwise comparisons using Tukey-adjusted contrasts to compare performance between attempts, within each group. Estimated marginal means significantly improved across consecutive attempts for both groups, except for the attempts three to four, *t*(1315) = 2.04, *p* = 0.321, for enlisted; *t*(1315) = 2.22, *p* = 0.230 for NFS, and attempts five to six, *t*(1315) = 2.20, *p* = 0.240 for enlisted; *t*(1315) = 2.06, *p* = 0.308 for NFS. Unlike VTT, NFS performed significantly better than enlisted across all six ATT attempts.

Overall ATT improvements across the six attempts were large for both groups (see Table 2). In terms of Cohen’s *d* improvements, enlisted improved a total of *d* = 1.80 and NFS improved *d* = 1.25. Both groups also experienced the largest gains between the first and second attempt, *d* = 0.66 for enlisted and *d* = 0.53 for NFS.

#### Improvement based on initial performance

In a subsequent analysis, we assessed individual differences in learning rate on the two tracking tasks, specifically whether participants with lower initial performance improved more across attempts than those with higher initial performance. This analysis used mixed-effects growth curve modeling to evaluate the correlation between the random intercept (initial performance) and the random slope (rate of improvement), in which a positive correlation would indicate that better initial performers improved at a faster rate, and a negative correlation would indicate the opposite. To test the significance of each model’s intercept–slope correlation, we compared the unconstrained model (allowing intercepts and slopes to correlate) with a constrained model (not allowing the intercepts and slopes to correlate) using a likelihood ratio test following the approach of Bates et al. (2015).

**Fig. 4:**
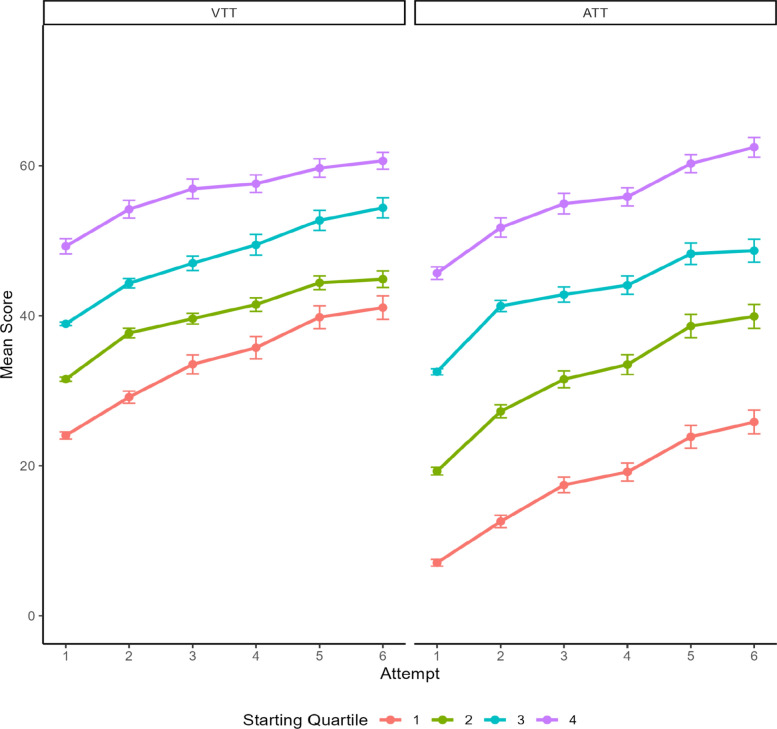
VTT and ATT Attempt Scores by Initial Performance Quartile. *Note*. Figure plots mean scores by attempt based on quartile of initial performance (i.e., first attempt) across the full sample (*N* = 265) for the VTT (left) and ATT (right). Error bars represent standard error of the mean

For the VTT, this correlation was *r* = −0.18, and the likelihood ratio test was significant (χ2 = 6.52, *p* = 0.01), indicating that initially worse performers had a slightly quicker rate of improvement, although this effect was small. For the ATT, this correlation was *r* = −0.08 and the likelihood ratio test was not significant (χ2 = 1.45, *p* = 0.23), indicating no relationship between initial performance and rate of improvement. To visualize rate of improvement by initial performance, Figure 4 shows performance across attempts broken down by initial performance quartiles for the VTT and ATT. Consistent with the results of the growth curve model, the VTT appears to have a slightly steeper rate of improvement for the first quartile, and the ATT had similar of improvement across the initial performance quartiles.

##### Performance stability

To test performance stability in the tracking tasks across attempts, we assessed both Spearman rank correlations and intraclass correlation coefficients (ICCs). The model used to calculate ICCs was the two-way mixed-effects model with absolute agreement, based on guidance from Koo and Li (2016). This model emphasizes the need for individuals to achieve the same score across repeated measurements and penalizes changes in score even if rank ordering is consistent. Therefore, Spearman rank correlations were informative as to whether rank ordering was consistent, whereas the ICC values were informative as to whether overall scores were consistent. These are presented in Tables 3 and 4.

**Table 3:** VTT test–retest reliabilities

Attempt	1	2	3	4	5	6
1	–	0.80	0.62	0.49	0.40	0.35
2	0.87	–	0.87	0.68	0.59	0.53
3	0.79	0.91	–	0.80	0.74	0.68
4	0.73	0.77	0.81	–	0.90	0.85
5	0.70	0.75	0.79	0.92	–	0.94
6	0.68	0.73	0.77	0.90	0.94	–

*N* = 265. Below the diagonal are Spearman rank correlations; above the diagonal are ICCs

**Table 4:** ATT test–retest reliabilities

Attempt	1	2	3	4	5	6
1	–	0.86	0.73	0.67	0.53	0.48
2	0.94	–	0.93	0.85	0.74	0.69
3	0.88	0.94	–	0.91	0.84	0.80
4	0.85	0.89	0.92	–	0.92	0.88
5	0.81	0.87	0.90	0.96	–	0.96
6	0.78	0.83	0.87	0.93	0.97	–

*N* = 265. Below the diagonal are Spearman rank correlations; above the diagonal are ICCs

Both Spearman correlations and ICC values were quite strong for *consecutive* attempts, ranging from ρ = 0.80–0.94 and ICC = 0.80–0.94 for VTT (Table 3) and ρ = 0.92–0.97 and ICC = 0.86–0.96 for ATT (Table 4). In line with our hypothesis, the weakest reliability values were between the first and final attempts for each task: ρ = 0.68 and ICC = 0.35 for VTT and ρ = 0.78 and ICC = 0.48 for ATT, indicating both shifting in rank ordering and that scores were not consistent from the first attempt to the sixth.

Table 5 shows the test–retest reliability between the VTT and ATT composite scores of their official administration of the ASTB as well as each PBM attempt in the present study. The mean amount of time between last taking the official ASTB and participating in this laboratory-based study was 779 days (*SD* = 418). For VTT, the first attempt of our study’s PBM correlated with the official administration at ρ = 0.70 and ICC = 0.50 and dropped to ρ  = 0.48 and ICC = 0.44 by the sixth attempt. For ATT, the first attempt of our study’s PBM correlated with the official administration at ρ  = 0.69 and ICC = 0.61 and dropped to ρ = 0.50 and ICC = 0.38 by the sixth attempt. The correlations for attempt one vs. attempt six to official PBM scores were significantly different from each other according to Steiger’s (1980) test for dependent correlations (see Hoerger, 2013), except the ICC for VTT (i.e., 0.50 vs. 0.44 was not a statistically significant decrease in ICC).

**Table 5:** Test–retest reliabilities for official test scores with study retest scores

	Study attempt number												
	1		2		3		4		5		6		
	ρ	ICC	ρ	ICC	ρ	ICC	ρ	ICC	ρ	ICC	ρ	ICC	
Official VTT	0.70	0.50	0.66	0.60	0.57	0.54	0.58	0.54	0.54	0.51	0.48	0.44	
Official ATT	0.69	0.61	0.61	0.59	0.55	0.51	0.55	0.47	0.54	0.43	0.50	0.38	

*ρ* = Spearman rank correlations; IC = intraclass correlation coefficients, calculated as two-way mixed model with absolute agreement. We were unable to link official ASTB scores for 3 NFS, resulting in an *n* = 143. For participants who took the official ASTB/PBM more than once, their most recent score was used

##### Predicting asymptotic performance

Even though the rate of improvement analyses indicated that performance on both VTT and ATT attempts six were not significantly better than on attempt five, we questioned whether participants were truly approaching their peak performance in these tasks. Our skepticism was based on the numerical (but not statistically significant) improvement in VTT and ATT composite scores from the fifth and sixth attempts as well as visual inspection of the overall gains across attempts. This motivated us to conduct Bayesian nonlinear generalized mixed-effects modeling to assess participant learning trajectories and estimate their asymptotic levels of performance (see Cochrane, 2020; Cochrane et al., 2023; and Kattner et al., 2017 for more information on this approach). This approach required aggregating z-scored VTT and ATT performance into a single metric and also to model performance on a more continuous level than the composite scores used as the dependent variable elsewhere throughout the analysis. As such, the models involved calculating the (square root-transformed, to reduce skew) mean distance participant cursors were from the target in 0.5-s increments while performing the tracking tasks, which for these analyses will be referred to as distance to target.

Participant-level trajectories were parameterized as an exponentially decaying magnitude of normalized distance to target due to exponential functions’ tendency to well characterize individuals’ learning trajectories (Cochrane & Green, 2021; Dosher & Lu, 2007; Heathcote et al., 2000). All models showed satisfactory convergence.^3^ From these models, we extracted quantities of change in distance (i.e., ending distance minus starting distance) as well as the amount of time necessary for learning to saturate (i.e., number of blocks until improvement reaches 90% of the way from starting to asymptotic performance).

Quantitative fits to learning trajectories corroborated the large and protracted learning we observed in performance aggregated by attempt (see Tables 1 & 2); distances decreased by approximately -22.57 (equivalent to -3.99 standard deviations of starting distance values) and were still changing through the end. In fact, when assessing the by-participant amounts of training necessary for learning to saturate we found that it was far beyond the six attempts provided in the present study (median = 15.29 blocks). These results show that improvements were continuing throughout the task, but they also indicate that estimates of asymptote are likely to be imprecise due to the small amount of data relative to the long times to learn. That is, although inter-individual estimates of asymptotic performance and rate of change are unlikely to be highly reliable, the group-level pattern of results showed an extended trajectory of training-related improvements. To quantify this more straightforwardly, our analytic method revealed that the average participant was only 62% of the way to asymptotic performance, the theoretical point of stability. To achieve 90% of the asymptotic performance (i.e., saturation level), these analyses predicted an average of 16 (*SD* = 8.51) attempts on each task would be required.

We intended to perform additional follow-up analyses pertaining to these learning trajectories; however, these were abandoned upon seeing that the results of the initial modeling analyses indicated that participants were still quite early in the learning process. Because of this, further modeling results would likely be volatile and unreliable.

### Research question 2: influence of gaming experience

#### Descriptive statistics

Our second research question was the potential influence of relevant gaming experience on VTT and ATT performance. Table 6 shows the overall descriptive statistics, and Table 7 shows the descriptive statistics broken up by group. Note that 15 participants did not take the background survey, resulting in an *n* of 250 for these analyses (106 enlisted; 144 NFS).

**Table 6:** Descriptive statistics for gaming experience

Gaming variable	*M*	*SD*	Skew	Kurtosis
Childhood Action	7.30	4.79	0.26	−0.49
Adult Action	5.30	4.63	0.50	−0.81
Childhood Flight Sim	0.78	1.36	1.81	2.37
Adult Flight Sim	0.62	1.18	2.27	4.90

*n* = 250 (106 enlisted; 144 NFS). Action gaming experience was the sum of 0–5 responses across the four relevant video gaming genres of the gaming survey and thus scores could range from 0 to 20. Flight simulator gaming experience was a specific question on the survey and thus scores could range from 0 to 5. Participants answered separate questions for adult (within the last 12 months) and childhood (between the ages of 3 and 13 years) levels of gaming engagement

**Table 7:** Descriptive statistics for gaming experience by group

Gaming variable	Enlisted				NFS			
	*M*	*SD*	Skew	Kurtosis	*M*	*SD*	Skew	Kurtosis
Child Action	7.72	5.03	0.33	−0.29	6.99	4.60	0.17	−0.79
Adult Action	6.35	4.82	0.27	−0.96	4.53	4.34	0.66	−0.61
Child Flight Sim	0.58	1.21	2.39	5.40	0.92	1.44	1.51	1.23
Adult Flight Sim	0.35	1.05	3.62	12.97	0.82	1.23	1.71	2.63

*n* = 250 (106 enlisted; 144 NFS). Action gaming experience was the sum of 0–5 responses across the four relevant video gaming genres of the gaming survey and thus scores could range from 0 to 20. Flight simulator gaming experience was a specific question on the survey and thus scores could range from 0 to 5. Participants answered separate questions for adult (within the last 12 months) and childhood (between the ages of 3 and 13 years) levels of gaming engagement

Action video gaming responses had good variability and were roughly normally distributed with skewness and kurtosis values within ± 1 (both in the combined data and when broken down by group). However, responses for the childhood and adult flight simulator gaming frequency were quite low (with a mean below 1) and non-normal, especially for the enlisted group. Specifically, 67% of participants reported never playing flight simulator games in childhood and 70% reported never playing any as an adult (i.e., within the last 12 months). For enlisted, these numbers were especially high, at 74% for childhood and 85% for adult. There would be good justification to exclude the flight simulator gaming variables from subsequent analysis, but we decided to retain them with the strong caveat that there is not much variability or normality in the distribution of responses.

#### Correlational analysis

Due to normality concerns with the flight simulator gaming frequency variables, and at the recommendation of a reviewer, we conducted Spearman’s rank-order correlation coefficient (a nonparametric measure) to assess the relationship between self-reported gaming frequency and tracking performance on each VTT and ATT attempt. Table 8 shows these correlations for the entire sample, and Table 9 shows them separated by group membership.

**Table 8:** Spearman correlations with background variables across attempts

Task	Attempt	Child action	Adult action	Child flight	Adult flight
**VTT**					
	1	**0.14**	**0.14**	0.09	**0.21**
	2	**0.14**	**0.14**	0.04	**0.17**
	3	**0.18**	**0.15**	0.09	**0.19**
	4	**0.24**	**0.19**	0.11	**0.16**
	5	**0.24**	**0.23**	0.09	**0.15**
	6	**0.21**	**0.19**	0.07	**0.13**
**ATT**					
	1	**0.15**	0.06	**0.17**	**0.32**
	2	**0.14**	0.10	**0.13**	**0.28**
	3	**0.17**	**0.13**	0.11	**0.26**
	4	**0.17**	0.10	0.11	**0.26**
	5	**0.18**	**0.13**	0.10	**0.23**
	6	**0.19**	0.12	0.10	**0.22**

*n* = 250. Boldface indicates statistical significance at *p* < 0.05

**Table 9:** Spearman correlations with gaming variables across attempts broken by group

Task	Attempt	Enlisted (*n* = 106)				NFS (*n* = 144)			
		Child action	Adult action	Child flight	Adult flight	Child action	Adult action	Child flight	Adult flight
**VTT**									
	1	0.00	0.00	0.04	−0.03	**0.28**	**0.37**	0.09	**0.27**
	2	−0.03	−0.05	−0.02	−0.02	**0.32**	**0.37**	0.04	**0.24**
	3	0.00	−0.06	0.00	0.00	**0.36**	**0.40**	0.11	**0.25**
	4	0.10	−0.01	0.06	0.01	**0.38**	**0.42**	0.12	**0.17**
	5	0.09	0.01	0.03	0.02	**0.37**	**0.45**	0.11	**0.19**
	6	0.07	0.00	0.06	0.05	**0.34**	**0.38**	0.06	**0.17**
**ATT**									
	1	0.13	0.11	0.08	0.08	**0.27**	**0.26**	0.10	**0.23**
	2	0.08	0.10	0.09	0.09	**0.29**	**0.34**	0.04	**0.19**
	3	0.10	0.10	0.06	0.06	**0.31**	**0.31**	0.04	**0.19**
	4	0.12	0.06	0.06	0.06	**0.29**	**0.31**	0.05	**0.19**
	5	0.11	0.07	0.07	0.07	**0.30**	**0.33**	0.04	**0.19**
	6	0.12	0.09	0.09	0.09	**0.29**	**0.32**	0.03	**0.17**

Boldface indicates statistical significance at *p* < 0.05

#### Action video gaming experience and tracking performance

In the combined dataset from Table 8, we can see that childhood action gaming frequency was significantly, but fairly weakly, correlated with both VTT and ATT across all attempts, ranging from ρ = 0.14–0.24. Correlations involving adult action gaming levels were more sporadic, with significant correlations with performance on all VTT attempts (ρ = 0.14–0.23) but only attempts 3 and 5 of ATT (ρ = 0.13). There was a slight trend for correlations for the second session (i.e., attempts 4–6) to be slightly numerically stronger than the first session (attempts 1–3), possibly hinting that participants with more gaming experience improved more than those with less experience. Breaking these down by group membership (Table 9) revealed that these correlations were driven by NFS participants, as no correlations involving enlisted participants and action gaming frequency were significant for any VTT or ATT attempt among enlisted participants, whereas for NFS both childhood and adult action gaming were significantly correlated with performance on all VTT and ATT attempts. Specifically, for NFS, childhood action gaming frequency was moderately correlated with VTT performance for all attempts (ρ = 0.28–0.38) with relatively consistent correlations across attempts except for attempt 1. Similarly, adult action gaming was moderately to strongly correlated with VTT performance, ranging from ρ = 0.37–0.45. For ATT performance, correlations ranged from ρ = 0.27–0.31 with childhood action gaming and ρ = 0.26–0.34 for current action gaming, and correlations were generally numerically consistent across attempts.

#### Flight simulator gaming experience and VTT/ATT performance

Despite the aforementioned psychometric issues with the self-reported flight simulator gaming experience variables, we found some significant correlations involving flight simulator frequency and VTT or ATT performance Specifically, in the combined dataset consisting of both enlisted and NFS participants, self-reported child flight simulator gaming frequency correlated significantly (but weakly) with VTT performance on attempts 1 and 2 (ρ = 0.17 and .13, respectively), and adult flight simulator gaming frequency correlated significantly with performance on all attempts of both VTT (ρ = 0.13–0.21) and ATT (ρ = 0.22–0.32). The trend for the correlations involving flight simulator gaming was for numerically weaker correlations across attempts, possibly indicating that participants with less flight simulator experience improved more quickly on the tracking tasks. When broken down by group membership, Table 9 reveals that the significant correlations involving tracking performance and flight simulator experience in the combined dataset were primarily driven by NFS, as there were no significant correlations involving enlisted participants. Among NFS, child flight simulator frequency was not significantly correlated with any VTT or ATT attempts, but adult flight simulator levels were weakly to significantly correlated with all attempts both the VTT (ρ = 0.17–0.25) and ATT (ρ = 0.17–0.23). There was a slight numerical trend for stronger correlations for earlier attempts, potentially indicating that individuals who currently played fewer hours of flight simulator games improved more quickly on the tracking tasks.

##### Linear mixed-effects models

To more formally test the effect of gaming experience on tracking performance, we performed linear mixed-effects regression analyses on both the combined dataset and the group-specific datasets (i.e., Enlisted, NFS). The models included participants as a random effect, and fixed effects of all gaming experience variables, attempt number, and their interactions (see Tables 10, 11, 12). Separate models were run for performance on the VTT and ATT. The focus of these models was the interaction of attempt number and gaming experience to test our hypothesis that participants with less gaming experience would benefit more from practice.

**Table 10:** Mixed-effects models for attempt number and background variables, full sample

Effect	VTT			ATT		
	β	*SE*	*t*	β	*SE*	*t*
Attempt Number	**0.35**	0.01	**31.99**	**0.32**	0.01	**39.10**
Childhood Action Gaming	0.13	0.07	1.97	**0.14**	0.070	**2.07**
Adult Action Gaming	0.04	0.07	0.55	−0.05	0.07	−0.78
Childhood Flight Sim	−0.01	0.06	−0.22	−0.02	0.06	−0.32
Adult Flight Sim	0.10	0.06	1.66	**0.26**	0.06	**4.14**
Childhood Action Gaming x Attempt Number	**0.03**	0.02	**2.40**	**0.04**	0.01	**3.52**
Adult Action Gaming x Attempt Number	**0.04**	0.02	**2.68**	0.00	0.01	0.37
Childhood Flight Sim. x Attempt Number	0.02	0.01	1.37	**−0.02**	0.01	**−2.08**
Adult Flight Sim. x Attempt Number	**−0.05**	0.01	**−3.58**	−0.02	0.01	−1.90

*n* = 250. Boldface indicates statistical significance at *p* < 0.05

**Table 11:** Mixed-effects models for attempt number and background variables, enlisted only

	VTT			ATT		
Effect	β	*SE*	*t*	β	*SE*	*t*
Attempt Number	**0.39**	0.020	**20.85**	**0.41**	0.01	**28.88**
Childhood Action Gaming	0.07	0.11	0.64	0.08	0.11	0.74
Adult Action Gaming	−0.04	0.11	−0.33	−0.02	0.11	−0.21
Childhood Flight Sim	0.04	0.10	0.35	0.02	0.10	0.18
Adult Flight Sim	−0.01	0.10	−0.11	**0.22**	0.10	**2.17**
Childhood Action Gaming x Attempt Number	**0.06**	0.03	**2.40**	**0.06**	0.02	**3.06**
Adult Action Gaming x Attempt Number	−0.03	0.03	−1.16	−0.03	0.02	−1.78
Childhood Flight Sim. x Attempt Number	−0.01	0.02	−0.48	−0.02	0.02	−0.85
Adult Flight Sim. x Attempt Number	0.02	0.02	0.63	0.00	0.02	−0.08

*n* = 106. Boldface indicates statistical significance at *p* < 0.05

**Table 12:** Mixed-effects models for attempt number and background variables, NFS only

	VTT			ATT		
Effect	β	*SE*	*t*	β	*SE*	*t*
Attempt Number	0.**34**	0.01	**24.91**	**0.32**	0.01	**27.21**
Childhood Action Gaming	0.16	0.08	1.94	**0.19**	0.09	**2.10**
Adult Action Gaming	**0.20**	0.09	**2.34**	0.15	0.09	1.71
Childhood Flight Sim	−0.02	0.08	−0.19	−0.05	0.08	−0.58
Adult Flight Sim	0.09	0.08	1.14	0.12	0.08	1.44
Childhood Action Gaming x Attempt Number	0.02	0.02	1.23	**0.03**	0.02	**2.11**
Adult Action Gaming x Attempt Number	**0.08**	0.02	**4.52**	0.01	0.02	0.30
Childhood Flight Sim. x Attempt Number	**0.04**	0.02	**2.56**	−0.03	0.01	−1.78
Adult Flight Sim. x Attempt Number	**−0.08**	0.02	**−4.91**	−0.01	0.01	−0.58

*n* = 144. Boldface indicates statistical significance at *p* < 0.05

In the combined dataset from Table 10, the models revealed that attempt number had an expectedly large main effect for both VTT (β = 0.35) and ATT (β = 0.32), indicating that participants improved significantly across attempts. Our primary focus was the interaction between attempt number and each of the four gaming experience variables, with a positive β indicating participants with more gaming experience tended to improve more quickly across the six attempts, and a negative *β* indicating that people with less gaming experience improved more quickly. For VTT performance, both childhood and adult action video gaming frequency had significant, positive, interactions with attempt number (β = 0*.*03 and 0.04, respectively)*.* Childhood flight simulation gaming frequency did not interact with attempt number, and adult flight simulation frequency had a significant negative interaction with attempt number (β = −0.05) such that participants with less adult flight simulation gaming experience improved more quickly. For ATT performance, only childhood action gaming (β = 0.04) and childhood flight simulation gaming frequency (*β* = −0.02) interacted with attempt number. Specifically, participants with more childhood action video gaming experience improved more quickly across the six ATT attempts, whereas participants with more childhood flight simulation gaming experience improved more slowly relative to participants with less experience.

For the models restricted to only enlisted participants (Table 11), attempt number again had an expectedly large effect (β = 0*.*39 for VTT and β = 0*.*41 for ATT). For VTT performance, only childhood action gaming significantly interacted with attempt number (β = 0*.*06), indicating that enlisted participants with more childhood action gaming experience improved more quickly on the VTT relative to enlisted participants with less experience. This was the same for ATT performance, with the only significant interaction with attempt number being childhood action gaming (β = 0*.*06).

For the models restricted to only NFS participants (Table 12), the pattern of results was less straightforward. The main effect of attempt number was positive and significant as expected (β = 0*.*34 for VTT and β = 0*.*32 for ATT), and for VTT performance adult (i.e., current) action gaming (β = 0.08), childhood flight simulator gaming (β = 0.04), and adult flight simulator gaming levels (β = −0.08) all interacted with attempt number. Participants with more adult action gaming and childhood flight simulator gaming experience improved more across attempts relative to those with less experience, whereas participants with more adult flight simulator experience improved less across attempts relative to those with less experience. For performance on the ATT, only childhood action gaming frequency significantly, and positively, interacted with attempt number (β = 0*.*03).

### Research question 3: predictive validity

To assess whether there was differential predictive validity of VTT and ATT performance, we compared the relative strength of correlations of composite scores on attempts one through six to flight grades from the Naval Introductory Flight Evaluation (NIFE) program. According to the US Navy, NIFE is designed to establish “…the foundations of aviation fundamentals for aspiring aviators but is also a screening tool that will test students’ ability to handle stressful evolutions in a high impact environment” (America’s Navy, 2024). NIFE is the initial step of Naval aviation training lasting approximately two months and broken down into four phases: water survival training (one week), academics (three weeks), flight performance (variable length), and aviation physiology (one week). During the flight performance phase, “…students must quickly memorize and prioritize information required to complete a series of flights in a Cessna 172 using Navy flight procedures…students are expected to attempt at least six landings and perform set of standardized maneuvers on each of seven flights.” (American’s Navy, 2024). At the end of this flight phase, students receive a performance score, which is re-normed every 200 students to a mean of 50 and standard deviation of 10. Note that NIFE training is exclusive to NFS. We did not have a standardized outcome to use for predictive validity analyses for our enlisted participants as they came from multiple ratings (occupations) and thus had widely varied experiences in their respective training programs. Also note that we had intended to include outcome scores further down in the NFS training pipeline (i.e., post-NIFE flight school), but due to factors out of our control a large portion of our NFS participants were selected for an experimental version of primary flight school training which would complicate analyses and comparisons.

All six of the VTT and ATT attempts exhibited restriction of range relative to a multiyear sample of 43,883 applicant scores (VTT: *SD* = 16.70; ATT: *SD* = 18.77) presented in Sibley (2024). For the VTT, this yielded a restriction ratio ranging from *u* = 0.67 to *u* = 0.83 and for the ATT, *u* = 0.81 to *u* = 0.96, from attempt 1 to 6. Table 13 shows the correlation of NIFE flight scores to VTT and ATT composite scores across each attempt. Values within parentheses represent corrected correlations, calculated using Thorndike’s (1949) Case 2 for explicit selection. Also included for comparison are the correlations of VTT and ATT scores to each participant’s official ASTB administration, that is the one they took prior to the present study as part of the selection process for beginning NIFE training. Interestingly, and for reasons that are not immediately apparent, the predictive validity of the VTT and ATT composite scores were essentially flipped from the official administration such that in our data VTT scores significantly correlated with NIFE flight grades (*r* = 0.24–0.32) whereas ATT scores did not (*r* = 0.06–0.10, *ns*). That aside, our main question was whether predictive validity would change across attempts, and the correlations indicated that this is not the case as Steiger’s (1980) test of dependent correlations (see Hoerger, 2013) revealed the correlations between NIFE flight grades and attempt one vs. attempt six were not significantly different from one another for either VTT or ATT.

**Table 13:** Pearson correlations between VTT and ATT composite scores and NIFE flight performance

	Official test attempt	Study attempt number					
	Most recent	1	2	3	4	5	6
VTT	0.15 (0.14)	**0.24 (0.35)**	**0.21 (0.29)**	**0.30 (0.39)**	**0.32 (0.39)**	**0.30 (0.37)**	**0.27 (0.33)**
ATT	**0.22 (0.24)**	0.09 (0.13)	0.10 (0.13)	0.06 (0.07)	0.09 (0.11)	0.09 (0.11)	0.09 (0.11)

Ten of the 146 NFS participants dropped out before completing NIFE Flight training. Additionally, we were unable to link three participants’ NIFE flight scores with their scores from the present study, resulting in an *n* of 133 for these analyses. Boldface indicates *p* < 0.05, and correlations within parentheses are corrected to account for restriction of range

**Fig. 5:**
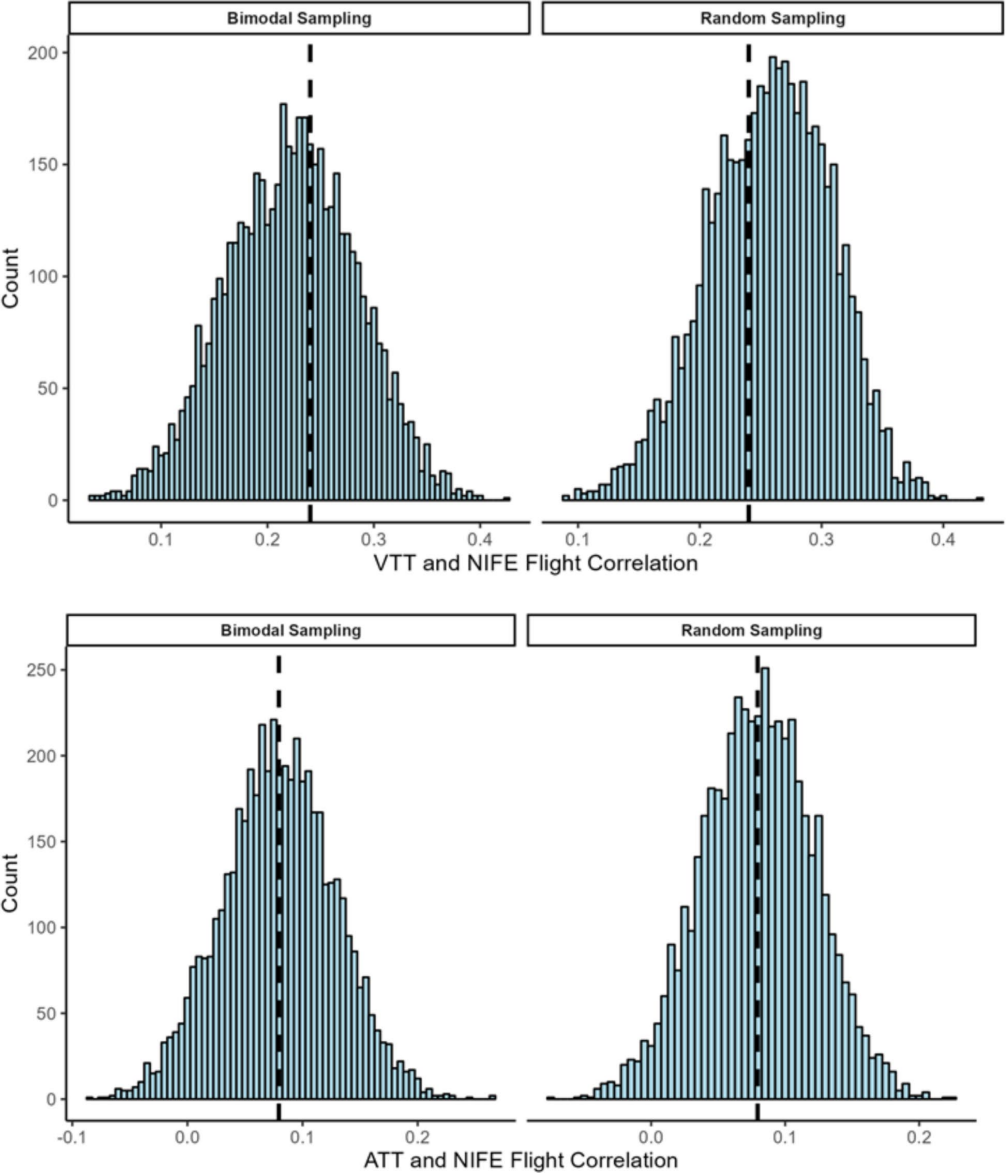
Predictive Validity of VTT and ATT Across the 5,000 Practice Distribution Simulations. *Note*. Results are based on 5,000 iterations each

We were also interested in the extent to which predictive validity of these tracking tasks was impacted by participants having differing levels of practice with them. To assess this, we compared two different practice distribution patterns separately for each task to answer this question: one in which we randomly selected any one score from each participant’s six attempts (random sampling), and one in which we randomly selected with equal probability either the first or sixth attempt for each participant (bimodal sampling; see Burgoyne et al., 2025). Each method produced one task score per participant from one of their six attempts. We then calculated the correlation between that set of scores and NIFE flight performance and then repeated the process for a total of 5,000 iterations for each tracking task and sampling distribution. This provided a distribution of correlations, shown in Table 14 and Fig. 5. The results show that the average correlation between VTT performance and NIFE flight performance was *r* = 0.26 (random sampling) and *r* = 0.22 (bimodal sampling), with 95% of the correlations falling within *r* = 0.15–0.35 (random sampling) and *r* = 0.11–0.34 (bimodal sampling). For ATT, the average correlation with NIFE flight performance was *r* = 0.08 for both sampling methods, with 95% of the correlations falling within *r* = 0.00–0.16 (random sampling) and *r* = -0.02–0.17 (bimodal sampling).

**Table 14:** Descriptive statistics for predictive validity distribution of practice distribution simulations

Task	Sampling distribution	*M (SD)*	95% Range	Min	Max	Skew	Kurtosis
VTT	Random	0.26 (0.05)	0.15–0.35	0.09	0.43	−0.21	−0.17
	Bimodal	0.22 (0.06)	0.11–0.34	0.03	0.42	−0.05	−0.26
ATT	Random	0.08 (0.04)	0.00–0.16	−0.07	0.22	−0.05	−0.1
	Bimodal	0.08 (0.05)	−0.02–0.17	−0.09	0.26	−0.02	−0.06

Results are based on 5,000 iterations each

## Discussion

### General learning effects and stability of individual differences in the PBM

The present study sought to address a number of questions relating to the effects of practice among both enlisted Navy personnel and Navy aviators in two psychomotor tracking tasks included in a military selection battery. Regarding our first research question as to the extent of improvements from practice, our results expectedly showed significant gains across PBM attempts for both tracking tasks of around *d* = 1.25 from attempts one to six. When broken by group, NFS expectedly performed better on both the VTT and ATT, but enlisted personnel improved more relative to NFS numerically (*d* = 1.56 vs. 1.27 from attempt one to six on VTT; *d* = 1.80 vs. 1.25 from attempt one to six on ATT). Consequently, enlisted vs. NFS performance was statistically equal on the fifth and sixth attempts of the simpler VTT, although NFS outperformed enlisted participants throughout all attempts on the more complex ATT. The relatively smaller gains for NFS aligned with our hypotheses given that NFS had already performed these tasks (and performed sufficiently well on them) as part of their selection process to become a Naval Flight Officer, and additionally many of them presumably had extensive practice from prior preparation efforts and therefore less room for improvement. These results are also consistent with Carretta et al. (2000) who administered the US Air Force’s Basic Attribute Test (strongly analogous to the PBM) twice to college students and found that the weakest initial performers improved the most, though in the present study growth curve modeling indicated that this was only true for the VTT and with a small effect size. This is a surprising finding because our experience and results of other studies on these tracking tasks strongly suggest that becoming familiar and comfortable with the y-axis inversion of the stick’s controls in the ATT is a significant determinant of performance, and so we expected here that initial poor performers would improve more as they became more comfortable with these controls.^4^ Relatedly, it is noteworthy that NFS still showed large improvements on these tracking tasks and that the SDs for performance increased across attempts for both tasks among both enlisted and NFS participants. This is counter to Ackerman’s (2022) observation that, in process tests of this ilk, “…between-individual variability typically shows a substantial reduction with increased learning/practice opportunities” (p. 9). As mentioned in Introduction, if post-practice performance on the tracking tests had significantly reduced between-subject variance as well as reduced correlations with variables of interest, then this could be a concern for using them for personnel selection.

Test–retest reliabilities between consecutive PBM attempts were strong but only around 46% of the performance variance in VTT and 61% in the ATT carried over from the first to sixth attempt. This means there was some degree of shuffling in the rank ordering of participants across the attempts, i.e., that participants improved at different rates. Spearman rank-order correlations between VTT and ATT performance in the in-laboratory attempts of the present study with the NFS official PBM scores (with an average retest interval of over two years) were initially around ρ = 0.70, which is close to the test–retest reliability that Carretta et al. (2000) found in the Air Force’s Basic Attributes Battery psychomotor scores when retesting after two weeks or three months. These values also expectedly decreased across each attempt such that we found only around 20% shared variance in tracking performance on the official test with the final attempt of our in-laboratory PBM. This similarly demonstrates shuffling in the rank ordering of individuals, which is interesting for a highly down-selected group. Overall, the pattern of these correlations suggests that there is room to explore and identify variables and effects that are contributing to some people improving at a quicker rate than others.

We hypothesized that participants would achieve close to asymptotic tracking performance by the sixth and final attempts of the in-laboratory PBM. On the one hand, we found that participants did not perform significantly better on either the VTT or ATT on their sixth attempt relative to the fifth. On the other, examination of their improvements indicated that they may not yet be at a leveling off point by the sixth attempt. This motivated us to perform post hoc Bayesian modeling analyses of their learning trajectories to estimate the location of their hypothetical asymptotic level of performance. These analyses indicated that our participants were on average still less than 2/3 their way through their VTT and ATT learning process by the end of the sixth PBM attempt, and that it would take a total of 16 attempts of our iterative PBM for them to reach 90% of their estimated asymptotic performance. Note, however, that these models were not particularly stable because participants were still so early in the learning process by the end of the present study that we did not have sufficient data to accurately model true asymptotic performance. That is, stable performance may occur much earlier or later than the model predicts. Either way, the results did clearly show that performance on these tracking tasks was not capped after six attempts.

To summarize the findings from our first set of research questions pertaining to improvement and performance stability, our results indicate that there are strong practice effects in these tracking tasks which are still occurring even after six administrations and there is a good degree of individual differences in the rate of improvement.

### Influence of gaming experience on PBM improvement rate

Our second research question concerned whether gaming experience contributed to improvements on the tracking tasks, and thus may partially explain the individual differences in learning rates. We predicted that those with less action and/or flight simulator video gaming experience would improve more given they presumably started off at a lower level due to their relative lack of experience and comfort with both HOTAS-like devices and engaging less in games with similar demands as the tracking tasks. We ultimately did not find strong evidence for this. While enlisted personnel did improve more on the tracking tasks than NFS, in the combined dataset (i.e., before breaking the data up by group membership), for the VTT only current flight simulator gaming engagement was negatively associated with improvement rate, and for ATT only childhood flight simulator engagement. Conversely, for the VTT both childhood and adult action gaming levels were positively associated with improvement rate, and for ATT this was true of childhood action gaming levels. This finding that participants who played more action video games generally improved more quickly on the tracking tasks tentatively supports findings and claims that action video gaming has a causal facilitative effect on learning in cognitive tasks (e.g., Zhang et al., 2021). Though this needs to be caveated in that overall effect sizes were fairly weak (β values were 0.03–0.04) and adult action gaming engagement was not associated with improvement rates across ATT attempts.

A closer analysis of the group-specific patterns involving PBM performance and background variables clarified that, for enlisted participants, the only background variable associated with improvement rate was childhood action gaming, such that individuals with more childhood action gaming experience improved more quickly on both the VTT and ATT. For NFS, adult action gaming levels were positively associated with VTT improvement rates, and childhood action gaming levels were also positively associated with ATT improvement rates, albeit weakly. Interestingly, childhood flight simulator gaming experience was positive associated with VTT improvement rate, but adult flight simulator gaming experience was negatively associated with VTT improvement rate. Neither childhood nor adult flight simulator levels were associated with improvement rates for the ATT.

The overall patterns of results indicate that action gaming levels tended to be positively associated with improvement rates, and effects were more sporadic for flight simulator experience but there was some evidence that individuals with less experience improved more quickly. We will not over interpret the results regarding flight simulator gaming experience due to the inconsistent pattern of effects and aforementioned issues with the non-normality and lack of variability in the flight simulator gaming responses. Our interpretation is therefore that relevant background variables do appear to have an association with tracking performance (which aligns with findings from Draheim et al., 2025; Drollinger et al., 2015, 2017; Ostoin, 2007), but there is a lack of strong evidence supporting our hypothesis that individuals with lower levels of relevant video gaming experience may benefit more from practice compared to more experienced gamers.

### Impact of practice on predictive validity of the PBM

Our third research question pertained to the more applied question of the predictive validity of the tracking portion of the PBM and how it was impacted by practice. Restricting the analysis to only NFS for whom we had comparable outcome data, we found that both VTT and ATT had stable prediction of NIFE flight grades across their attempts, with no significant difference in the relationship between NIFE flight grades and attempts one vs. six in these tasks. It is noteworthy and not readily explainable that the magnitude of the correlations between our six administrations of ATT and VTT with NIFE flight grades were essentially flipped from their official PBM administration. That is, our administrations of the VTT, but not ATT, were statistically significantly predictive of NIFE flight grades, whereas for their official scores ATT was predictive and VTT was not. While this is curious, our research question was whether prediction changed across attempts, and not the magnitude of the prediction itself.

Our final set of analyses was a simulation of real-world selection testing in which examinees arrive with differing, and unknown, levels of practice and familiarity with the tests. When administering selection batteries it is typically assumed that content of the tests and processes involved in performing them are novel to participants (see Ackerman, 2022), in other words that participants are naive to its questions and demands. But we know from our internal data and other sources that this is not the case. Our practice distribution analyses revealed that when randomly sampling from one of the six PBM attempts from each participant (or sampling only from the first and sixth attempts) across 5,000 iterations, the average predictive validity of the VTT and ATT was around the same as the individual attempts, indicating that the average predictive validity of these tasks is not overly impacted by examinees having differential levels of experience. But a closer examination of the range of prediction is warranted. Randomly choosing one of the PBM attempts to be a participant’s score resulted in a correlation between tracking performance and NIFE flight grades of anywhere from *r* = 0.09–0.43 for VTT and *r* = −0.07–0.22 for ATT. Or, if sampling only from attempts one and six (i.e., simulating having a bimodal distribution consisting of many examinees with little experience and many with extensive experience on the tasks), these values were *r* = 0.03–0.42 for VTT and *r* = −0.09–0.26 for ATT. This is quite a spread and indicates that when using these tracking tests as part of the personnel selection process, predictive validity may be artificially high or artificially low (and nonexistent) depending on the differential levels of practice the examinees have. This is perhaps the most noteworthy finding in the present study, though it does warrant mention that the range in which 95% of the simulated correlations between tracking performance and NIFE grades was a fair bit narrower at *r* = 0.15–0.35 (random sampling) and *r* = 0.11–0.34 (bimodal sampling) for the VTT and *r* = 0.00–0.16 (random sampling) and −0.02–0.17 (bimodal sampling) for the ATT.

## Limitations

Our results should be interpreted and contextualized with limitations in mind. Some limitations relating to specific analyses were already mentioned above, but we also wanted to highlight two more general ones which impacted the results of most of our analyses.

Methodologically, one limitation was the extent and timing of the practice attempts. Our participants performed the PBM a total of only six times across two sessions which were a week apart. More distributed practice across a longer time period would be expected to produce greater practice effects due to known effects such as consolidation and incubation. Further, although we hypothesized that six attempts would be sufficient for participants to be approaching their asymptotic level of performance, some indicators including follow-up analyses suggested that this was not the case, and instead participants were still relatively early in the overall learning process as their sixth attempt performance was only around 2/3 of their estimated asymptote and they would need around 12 additional PBM sessions to get to 90% estimated asymptotic performance. Multisession studies are quite challenging to conduct with active duty volunteers who are in the midst of their training. This challenge is compounded with longer sessions and/or long intervals between sessions, and so the present design was a balance of what would be ideal versus what would be logistically feasible with the population of interest.

Our final *N* of 265 participants with valid tracking performance data is ostensibly adequate for correlational analyses (e.g., 200 + is often a rule of thumb for basic correlation analyses; simulations from Schönbrodt & Perugini, 2013, suggest 250 is a reasonable benchmark for typical studies). However, our participants were from two distinct populations which necessitated separating out analyses by group membership, and as such many of our key analyses involved only between 100 and 150 participants. This is on the low end for any correlational analysis, particularly ones with smaller effect sizes which we encountered here likely due in part to testing down-selected groups of participants. In particular, the predictive validity and background variable analyses could have benefitted from larger sample sizes to obtain more robust statistics for some of the sensitive analyses. And while we did initially have 500 participants complete the first session, again due to challenges with active duty populations the number who completed the second session was significantly lower as we observed an attrition rate approaching 50%.

## Implications & future directions

One of the main findings of the present study was that inter-individual variance does not decrease with practice on the PBM, which is counter to what would be expected based on some theories of individual differences in skill acquisition (e.g., Ackerman, 1988). Relatedly, this was also counter to our hypothesis that individuals lacking certain background experience or characteristics such as video game and flight simulator experience would benefit more from practice, thus narrowing the gap between top and bottom performers. Instead, we found some evidence that individuals who played action video games not only started at a higher performance level but also benefited slightly more from practice. More research is therefore needed to better understand this convergence question in regard to psychomotor skill acquisition, perhaps with a more focused design to address this specific question. Importantly, however, these results do suggest that the PBM psychomotor tests are robust to practice and familiarity in the sense that there is sufficient performance variance present for them to be considered in the aviator selection process.

Arguably our most interesting finding pertained to the results of our simulation of real-world selection in which we analyzed predictive validity of tracking performance to NIFE flight grades using different PBM attempt scores for participants. Overall predictive validity was not strongly impacted, again indicating some robustness of these psychomotor tests to practice effects. However, the range of each iteration of this simulation displayed a wide spread, in some cases showing essentially no or even a negative correlation between tracking performance and eventual flight grades. This is a more concerning finding that ought to be considered in the selection process.

In summary, although the PBM tracking tasks display some robustness to practice effects, it is still the case that large practice effects continue to occur even after several administrations of the battery and that this appears to have some impact on predictive validity. It would therefore be worthwhile to further explore the determinants of learning in this task, perhaps even considering learning rate into the selection process. Further, an examination of the PBM as a selection tool with more extensive, perhaps even standardized, practice appears to be warranted.

## Conclusion

It can no longer be assumed in the modern internet era that applicants taking high-stakes selection tests are truly naive to them. It is therefore on both researchers and practitioners to take additional steps to consider this when developing tests and utilizing them for important personnel selection decisions. The present study examined the impact of practice and background variables on performance and predictive validity of a psychomotor tracking task battery called the PBM which is a component of the ASTB used for selecting military aviators and flight officers into initial training programs. We found both encouraging evidence that the battery was robust to practice effects and relevant background factors in some regards, and discouraging evidence that these factors may negatively impact predictive validity. Ultimately, the PBM appears to be a valuable part of the selection process but with potential room for improvement, and we recommend future research that provides participants with more extensive practice as well as consideration of official standardization of practice opportunities to prospective applicants.

## Data Availability

Pursuant to US Department of Defense and Navy policy, the data are not to be made publicly available as they contain information and scores concerning a controlled and actively used personnel selection test.

## Footnotes

1 Note the Air Force has a battery very similar to the PBM called the Test of Basic Aviation Skills that includes a horizontal tracking task (instead of vertical) and a two-dimensional tracking task like the ATT.

2 Mashburn et al. (2024) also found evidence that participants’ ability to more broadly control their attentional resources fully mediated the relationship between PBM performance and simulated work performance. Discussion of this finding goes beyond the scope of the paper, but it does tie in with the idea that PMA relies heavily on cognition.

3 We additionally compared the exponential learning model to an identical model which did not include any time-varying parameters (i.e., implemented stable performance estimates over time), and we confirmed that the learning model provided a quantitatively superior fit to the data than the stable performance model. When comparing the models using an efficient approximation to leave-one-out cross-validation (Vehtari et al., 2017), the difference in goodness of fit was very large (mean improvement in predictive likelihood: 2492.6; mean pointwise within-model improvement SE: 73).

4 This could be because our other datasets involving the ATT typically only have one retest instead of five as with the present study, and perhaps most participants become reasonably comfortable with the controls after just one exposure.
